# The 100 most cited papers on thymoma: a bibliometric analysis

**DOI:** 10.1186/s13019-023-02201-9

**Published:** 2023-04-07

**Authors:** Lei Liu, Jiaqi Zhang, Guige Wang, Ke Zhao, Chao Guo, Cheng Huang, Shanqing Li, Yeye Chen

**Affiliations:** Department of Thoracic Surgery, Peking Union Medical College Hospital, Shuaifuyuan No. 1, Wangfujing Street, Dongcheng District, Beijing, People’s Republic of China

**Keywords:** Thymoma, Bibliometric analysis, Citations, Thymic carcinoma, Treatment

## Abstract

**Objectives:**

The aim of this bibliometric analysis was twofold: to identify the 100 most cited research articles on thymoma and to highlight future research opportunities in light of past and current research efforts.

**Methods:**

The Web of Science database was queried to identify the 100 most cited articles on thymoma. Imformations relevant to scientific research were extracted and analyzed: first author, journal, impact factor, type of article, year of publication, country, organization and keywords.

**Results:**

The publication year of the top 100 most cited articles ranged from 1981 to 2018, and the number of citations ranged from 97 to 1182. Most of the included articles are original (75/100) and are mainly retrospective studies (52/75). The United States has the most published articles and citations, and the *Annals of Thoracic Surgery* is the most sourced journal (*n* = 16). Through VOSviewer analysis, high-density keywords mainly come from thymic carcinoma/invasive thymoma management, immune-related diseases, and laboratory research.

**Conclusions:**

To our knowledge, this is the first bibliometric study on thymoma. We found most of the top 100 most cited articles are original and retrospective research. The United States has the published and cited works. Presently, the hot keywords for thymoma research has gradually tilted towards immune-related diseases and laboratory research.

## Introduction

Thymoma is the most common mediastinum tumor, but it is still rare. In previous studies, thymoma was classified as a type of thymic epithelial tumor and was analyzed in conjunction with thymic carcinoma. With the continuous deepening of follow-up research, people have gradually found that thymoma and thymic carcinoma are two different neoplasms, and there are great differences in biological behavior, clinical manifestations, and treatment methods. As a result, many medical centers have conducted much research on thymoma. After the publication of the first literature on thymoma [1], the literature in this area became increasingly affluent.

Scientific analysis of influential literature in a field can help us better understand the hot spots and recent advances in that field. Bibliometrics is a statistical approach to assessing the impact of published literature on any field [2, 3]. Recently, there has been more and more research on bibliometrics in medicine, of which citation analysis is the most commonly used method [4–6]. Citation analysis can reflect the impact of literature in the field and tap the most influential literature to help us better understand the research hot spots and latest advances in the field.

There has been bibliometric analysis on thymic epithelial tumors [7]. However, to our knowledge, there is currently no bibliometric analysis specifically for thymoma. Therefore, considering the specificity of thymoma in thymic epithelial tumors, we conducted this study. This study aimed to identify and analyze the characteristics of the 100 most cited articles related to thymoma.

## Materials and methods

### Identifying the top 100 most cited articles

We identified articles through the Web of Science (WOS) database, considered one of the most popular and well-established resources for clinical researchers interested in citation analysis [8], in June 2022. Keywords included "thymoma," "thymoma surgery," "thymoma chemotherapy," "thymoma radiotherapy," "thymoma treatment," "thymoma management," "thymoma malignancies," "thymic epithelial tumors," with no limitation on time, abstract availability, study type, or research subjects. Articles were ranked based on the total citations received from the databases. Previous studies frequently combined thymoma and thymic carcinoma into thymic epithelial tumors. Therefore, we included studies that covered both thymic carcinoma and thymoma in the screening process and studies that specifically targeted thymoma.

### Article analysis

Three reviewers screened and extracted the statistical data from the included literature in this study. For the 100 most cited articles, the author, country, institution, year of publication, publication journal of each article, the latest 2021 journal impact factor (IF) released in 2022, keywords, and type of literature are summarized.

VOSviewer (Leiden University, Leiden, Netherlands) was used to construct networks: co-authorship analysis of countries/organizations/authors, co-citation analysis of journals, citation analysis of articles, and co-occurrence analysis of keywords. Further, the first 100 keywords of the frequency were presented in two visualizations (overlay and density visualization) of the co-occurrence analysis to identify key terms in thymoma research, including studies conducted entirely on thymoma, and studies that were predominantly thymoma or had specific analyses for thymoma, although covering other thymic epithelial tumours.

## Results

**Fig. 1:**
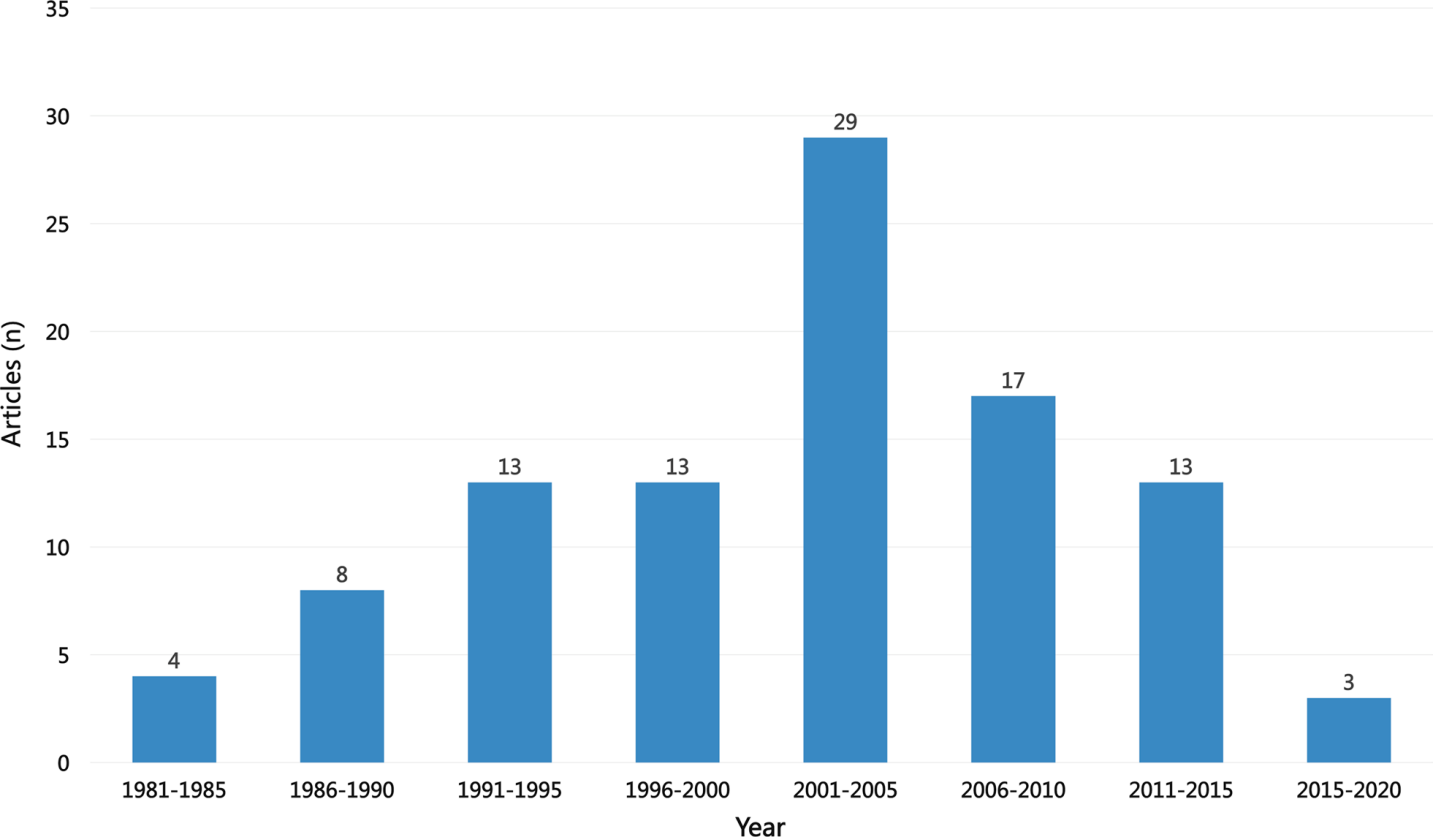
5-year interval for 100 most cited articles of thymoma

The top 100 most cited articles for thymoma studies were published from 1981 to 2018. The number of citations ranged from 97 to 1182, including a total of 17,049 citations as of June 2, 2022 (Table 1). One literature is cited more than 1000 times, and 22 pieces of literature are cited more than 200 times. When divided into five years, the period with the most significant distribution of literature was 2001–2005, with 29 published articles (Fig. 1). *Annals of Thoracic Surgery* accounted for the highest percentage of articles in the top 100 most cited articles, with 16 articles. According to the latest 2021 IF released in 2022, the top 5 journals are *Journal of Clinical Oncology, Journal of Thoracic Oncology*, *Clinical Cancer Research*, *Neurology* and *Annals of Neurology* (Table 2). The 100 most cited articles were categorized as 75 original articles, 19 reviews, 4 guideline/consensus/statement, 1 case report, and 1 communication/note (Table 3).

**Table 1:** The top 100 most cited articles on thymoma

Rank	First author	Title	Citation	Publication Year
1	Masaoka, A	Follow-up-study of thymomas with special reference to their clinical stages	1182	1981
2	Kondo, K	Therapy for thymic epithelial tumors: A clinical study of 1,320 patients from Japan	494	2003
3	Okumura, M	The World Health Organization histologic classification system reflects the oncologic behavior of thymoma—A clinical study of 273 patients	393	2002
4	Lewis, Je	Thymoma—a clinicopathological review	370	1987
5	Engels, EA	Malignant thymoma in the United States: Demographic patterns in incidence and associations with subsequent malignancies	322	2003
6	Marino, M	Thymoma and thymic carcinoma—relation of thymoma epithelial-cells to the cortical and medullary differentiation of thymus	317	1985
7	Regnard, JF	Prognostic factors and long-term results after thymoma resection: A series of 307 patients	304	1996
8	Koga, K	A review of 79 thymomas—modification of staging system and reappraisal of conventional division into invasive and noninvasive thymoma	295	1994
9	Blumberg, D	Thymoma—a multivariate-analysis of factors predicting survival	277	1995
10	Chen, G	New WHO histologic classification predicts prognosis of thymic epithelial tumors—A clinicopathologic study of 200 thymoma cases from China	274	2002
11	Engels, EA	Epidemiology of Thymoma and Associated Malignancies	270	2010
12	Verley, Jm	Thymoma—a comparative-study of clinical stages, histologic features, and survival in 200 cases	268	1985
13	Maggi, G	Thymoma—results of 241 operated cases	266	1991
14	Marx, A	The 2015 World Health Organization Classification of Tumors of the Thymus Continuity and Changes	263	2015
15	Detterbeck, FC	Thymic tumors	257	2004
16	Strobel, P	Tumor recurrence and survival in patients treated for thymomas and thymic squamous cell carcinomas: A retrospective analysis	224	2004
17	Detterbeck, FC	The IASLC/ITMIG Thymic Epithelial Tumors Staging Project: Proposal for an Evidence-Based Stage Classification System for the Forthcoming (8th) Edition of the TNM Classification of Malignant Tumors	217	2014
18	Kondo, K	WHO histologic classification is a prognostic indicator in thymoma	214	2004
19	Curran, Wj	Invasive thymoma—the role of mediastinal irradiation following complete or incomplete surgical resection	208	1988
20	Loehrer, Pj	Cisplatin plus doxorubicin plus cyclophosphamide in metastatic or recurrent thymoma—final results of an intergroup trial	204	1994
21	Kim, ES	Phase II study of a multidisciplinary approach with induction chemotherapy, followed by surgical resection, radiation therapy, and consolidation chemotherapy for unresectable malignant thymomas: final report	204	2004
22	Thomas, CR	Thymoma: State of the art	203	1999
23	Detterbeck, FC	The Masaoka-Koga Stage Classification for Thymic Malignancies Clarification and Definition of Terms	191	2011
24	Girard, N	Thymic epithelial tumours: ESMO Clinical Practice Guidelines for diagnosis, treatment and follow-up(aeuro)	182	2015
25	Quintanillamartinez, L	Thymoma—histologic subclassification is an independent prognostic factor	178	1994
26	Wright, CD	Predictors of recurrence in thymic tumors: Importance of invasion, World Health Organization histology, and size	174	2005
27	Kelleher, P	What is Good's syndrome? Immunological abnormalities in patients with thymoma	172	2003
28	Nakahara, K	Thymoma—results with complete resection and adjuvant postoperative irradiation in 141 consecutive patients	170	1988
29	Falkson, CB	The Management of Thymoma: A Systematic Review and Practice Guideline	170	2009
30	Marx, A	ITMIG Consensus Statement on the Use of the WHO Histological Classification of Thymoma and Thymic Carcinoma: Refined Definitions, Histological Criteria, and Reporting	168	2014
31	Nakagawa, K	Thymoma: A clinicopathologic study based on the new World Health Organization classification	165	2003
32	Fornasiero, A	Chemotherapy for invasive thymoma—a 13-year experience	161	1991
33	Tarr, PE	Infections in patients with immunodeficiency with thymoma (Good syndrome)—Report of 5 cases and review of the literature	161	2001
34	Loehrer, PJ	Cisplatin, doxorubicin, and cyclophosphamide plus thoracic radiation therapy for limited-stage unresectable thymoma: An intergroup trial	158	1997
35	Venuta, F	Long-term outcome after multimodality treatment for stage III thymic tumors	152	2003
36	Ruffini, E	Recurrence of thymoma: Analysis of clinicopathologic features, treatment, and outcome	149	1997
37	de Jong, WK	Thymic epithelial turnours: A population-based study of the incidence, diagnostic procedures and therapy	148	2008
38	Aarli, Ja	Patients with myasthenia-gravis and thymoma have in their sera igg autoantibodies against titin	147	1990
39	Loehrer, PJ	Combined etoposide, ifosfamide, and cisplatin in the treatment of patients with advanced thymoma and thymic carcinoma—An intergroup trial	142	2001
40	Giaccone, G	Cisplatin and etoposide combination chemotherapy for locally advanced or metastatic thymoma: A phase II study of the European Organization for Research and Treatment of Cancer Lung Cancer Cooperative Group	141	1996
41	Kelesidis, T	Good's syndrome remains a mystery after 55 years: A systematic review of the scientific evidence	141	2010
42	Monden, Y	Recurrence of thymoma—clinicopathological features, therapy, and prognosis	140	1985
43	Pan, CC	KIT (CDII7) is frequently overexpressed in thymic carcinomas but is absent in thymomas	135	2004
44	Sung, YM	F-18-FDG PET/CT of thymic epithelial tumors: Usefulness for distinguishing and staging tumor subgroups	135	2006
45	Masaoka, A	Staging System of Thymoma	135	2010
46	Ishibashi, H	Sex steroid hormone receptors in human thymoma	133	2003
47	Maggi, G	Thymomas—a review of 169 cases, with particular reference to results of surgical-treatment	133	1986
48	Hoffacker, V	Thymomas alter the T-cell subset composition in the blood: a potential mechanism for thymoma-associated autoimmune disease	133	2000
49	Venuta, F	Thymoma and thymic carcinoma	133	2010
50	Wilkins, KB	Clinical and pathologic predictors of survival in patients with thymoma	132	1999
51	Vernino, S	Autoantibody profiles and neurological correlations of thymoma	132	2004
52	Macchiarini, P	Neoadjuvant chemotherapy, surgery, and postoperative radiation-therapy for invasive thymoma	132	1991
53	Thomas, A	Sunitinib in patients with chemotherapy-refractory thymoma and thymic carcinoma: an open-label phase 2 trial	132	2015
54	Detterbeck, FC	Clinical value of the WHO classification system of thymoma	132	2006
55	Singhal, S	Comparison of stages I-II thymoma treated by complete resection with or without adjuvant radiation	128	2003
56	Suster, S	Thymoma, atypical thymoma, and thymic carcinoma—A novel conceptual approach to the classification of thymic epithelial neoplasms	128	1999
57	Lemma, GL	Phase II Study of Carboplatin and Paclitaxel in Advanced Thymoma and Thymic Carcinoma	127	2011
58	Zettl, A	Recurrent genetic aberrations in thymoma and thymic carcinoma	126	2000
59	Sommer, N	Myasthenic thymus and thymoma are selectively enriched in acetylcholine receptor-reactive t-cells	124	1990
60	Girard, N	Comprehensive Genomic Analysis Reveals Clinically Relevant Molecular Distinctions between Thymic Carcinomas and Thymomas	124	2009
61	Rea, F	Chemotherapy and operation for invasive thymoma	124	1993
62	Suster, S	Primary thymic epithelial neoplasms showing combined features of thymoma and thymic carcinoma—A clinicopathologic study of 22 cases	123	1996
63	Morgenthaler, Ti	Thymoma	123	1993
64	Scorsetti, M	Thymoma and thymic carcinomas	123	2016
65	Loehrer, PJ	Octreotide alone or with prednisone in patients with advanced thymoma and thymic carcinoma: An Eastern Cooperative Oncology Group phase II trial	121	2004
66	Petrini, I	A specific missense mutation in GTF2I occurs at high frequency in thymic epithelial tumors	120	2014
67	Radovich, M	The Integrated Genomic Landscape of Thymic Epithelial Tumors	120	2018
68	Meager, A	Anti-cytokine autoantibodies in autoimmunity: preponderance of neutralizing autoantibodies against interferon-alpha, interferon-omega and interleukin-12 in patients with thymoma and/or myasthenia gravis	119	2003
69	Tomiyama, N	Anterior mediastinal tumors: Diagnostic accuracy of CT and MRI	119	2009
70	Wilkins, Ew	Role of staging in prognosis and management of thymoma	118	1991
71	Okumura, M	Clinical and functional significance of WHO classification on human thymic epithelial neoplasms—A study of 146 consecutive tumors	118	2001
72	Sadohara, J	Thymic epithelial tumors: Comparison of CT and MR imaging findings of low-risk thymomas, high-risk thymomas, and thymic carcinomas	118	2006
73	Kaira, K	Biologic Correlation of 2-[F-18]-Fluoro-2-Deoxy-D-Glucose Uptake on Positron Emission Tomography in Thymic Epithelial Tumors	118	2010
74	Yamakawa, Y	A tentative tumor-node-metastasis classification of thymoma	117	1991
75	Jeong, YJ	Does CT of thymic epithelial tumors enable us to differentiate histologic subtypes and predict prognosis?	117	2004
76	Giaccone, G	Phase II Study of Belinostat in Patients With Recurrent or Refractory Advanced Thymic Epithelial Tumors	115	2011
77	Shelly, S	Thymoma and autoimmunity	114	2011
78	Buckley, C	Mature, long-lived CD4( +) and CD8( +) T cells are generated by the thymoma in myasthenia gravis	113	2001
79	Bernard, C	Thymoma associated with autoimmune diseases: 85 cases and literature review	113	2016
80	Willcox, N	Myasthenic and nonmyasthenic thymoma—an expansion of a minor cortical epithelial-cell subset	112	1987
81	Rea, F	Long-term survival and prognostic factors in thymic epithelial tumours	110	2004
82	Kim, DJ	Prognostic and clinical relevance of the World Health Organization schema for the classification of thymic epithelial tumors—A clinicopathologic study of 108 patients and literature review	110	2005
83	Gautel, M	Titin antibodies in myasthenia-gravis—identification of a major immunogenic region of titin	109	1993
84	Kondo, K	Lymphogenous and hematogenous metastasis of thymic epithelial tumors	109	2003
85	Katsuya, Y	Immunohistochemical status of PD-L1 in thymoma and thymic carcinoma	107	2015
86	Mygland, A	Ryanodine receptor autoantibodies in myasthenia-gravis patients with a thymoma	107	1992
87	Okumura, M	Results of surgical treatment of thymomas with special reference to the involved organs	106	1999
88	Margaritora, S	Thirty-Five-Year Follow-Up Analysis of Clinical and Pathologic Outcomes of Thymoma Surgery	106	2010
89	Pennathur, A	Comparison of surgical techniques for early-stage thymoma: Feasibility of minimally invasive thymectomy and comparison with open resection	106	2011
90	Marx, A	Thymoma and paraneoplastic myasthenia gravis	106	2010
91	Pescarmona, E	Analysis of prognostic factors and clinicopathological staging of thymoma	104	1990
92	Evoli, A	Thymoma in patients with MG—Characteristics and long-term outcome	104	2002
93	Girard, N	Thymoma A Focus on Current Therapeutic Management	104	2009
94	Ogawa, K	Postoperative radiotherapy for patients with completely resected thymoma—A multi-institutional, retrospective review of 103 patients	103	2002
95	Ruffini, E	Tumours of the thymus: a cohort study of prognostic factors from the European Society of Thoracic Surgeons database	103	2014
96	Chalabreysse, L	Correlation of the WHO schema for the classification of thymic epithelial neoplasms with prognosis—A retrospective study of 90 tumors	102	2002
97	Lee, EK	Morvan's fibrillary chorea: a paraneoplastic manifestation of thymoma	102	1998
98	Bodner, J	Early experience with robot-assisted surgery for mediastinal masses	100	2004
99	Wright, CD	Induction chemoradiotherapy followed by resection for locally advanced masaoka stage III and IVA thymic tumors	99	2008
100	Davenport, E	The role of surgery in the management of thymoma: A systematic review	97	2008

**Table 2:** Journals and their impact factors publishing more than 2 articles in the 100 most cited articles on thymoma

Rank	Journal	Articles	Citations	Impact factor
1	Annals of Thoracic Surgery	16	2793	5.102
2	Cancer	12	3453	6.921
3	Journal of Clinical Oncology	10	1619	50.717
4	Journal of Thoracic Oncology	8	1518	20.121
5	Journal of Thoracic And Cardiovascular Surgery	8	1298	6.439
6	European Journal of Cardio-Thoracic Surgery	3	346	4.534
7	Annals of Neurology	3	344	11.274
8	American Journal of Surgical Pathology	3	343	6.298
9	Lung Cancer	2	311	6.081
10	Clinical and Experimental Immunology	2	266	5.732
11	Clinical Cancer Research	2	256	13.801
12	American Journal of Pathology	2	238	5.77
13	European Journal of Radiology	2	237	4.531
14	Neurology	2	213	11.8

**Table 3:** Article type and study design composing the 100 most cited articles on thymoma

Article type	No. of articles
Original article	75
Original retrospective study	52
Original prospective study	12
Laboratory study	11
Review	19
Guideline/consensus/statement	4
Case Report	1
Communication/Note	1

Among authors of the top 100 most cited works, the top five are Monden Y, Masaoka A, Nakahara K, Tanioka T, and Loehrer Pj, with 2309, 1832, 1715, 1182, and 828 citations, respectively. Considering both the number of articles and the number of citations, the top five authors with the most published articles are Monden Y, Nakahara K, Loehrer Pj, Masaoka A, and Marx A, with 6, 5, 5, 4, and 4 articles (Table 4). The 100 most cited articles come from 17 countries. The top five countries are the U.S.A, Japan, Italy, England, France, and Germany (Table 5). The 100 most cited articles come from 198 organizations, with the top five being Osaka University, Tokushima University, National Cancer Institute, Memorial Sloan Kettering Cancer Center and Indiana University.

**Table 4:** Authors that contributed 3 or more articles in 100 most cited articles on thymoma

Rank	Author	Articles	Citations
1	Monden, Y	6	2309
2	Detterbeck, Frank C	6	1740
3	Nakahara, K	5	1715
4	Loehrer, Pj	5	828
5	Masaoka, A	4	1832
6	Marx, A	4	757
7	Girard, Nicolas	4	659
8	Giaccone, Giuseppe	4	584
9	Fujii, Y	4	481
10	Willcox, N	4	468
11	Kondo, K	3	817
12	Matsuda, H	3	617
13	Okumura, M	3	617
14	Nicholson, Andrew G	3	576
15	Casadio, C	3	548
16	Maggi, G	3	548
17	Van Schil, Paul	3	512
18	Livingston, R	3	504
19	Ruffini, Enrico	3	488
20	Muller-Hermelink, Hk	3	483
21	Aarli, Ja	3	363
22	Rea, F	3	395
23	Miyoshi, S	3	394

**Table 5:** Continents and countries of origin in the 100 most cited articles on thymoma

Continent & country	Articles	Citations
Europe	74	11,345
Italy	21	3006
England	12	1777
France	11	1850
Germany	11	2022
Belgium	6	889
Switzerland	4	716
Netherlands	3	401
Norway	3	363
Austria	2	209
Sweden	1	112
North America	46	7508
U.S.A	41	6786
Canada	5	722
Asia	32	6700
Japan	23	5175
China	5	946
South Korea	4	579
Middle-east	2	377
Israel	2	377
South America	1	120
Brazil	1	120

**Fig. 2:**
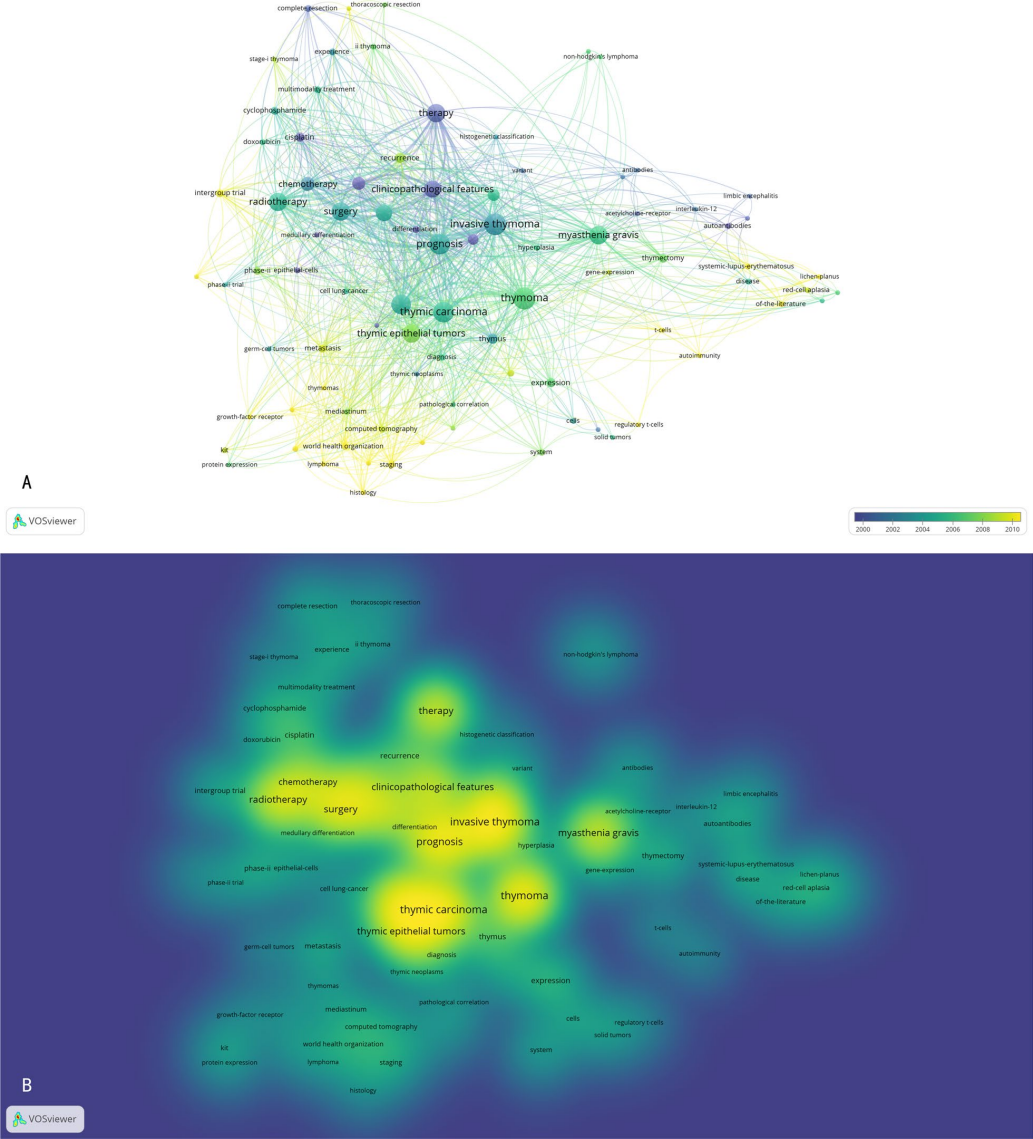
Co-occurrence analysis of keywords. **A** Distribution of keywords according to average publication year (blue: earlier, yellow: later). **B** Distribution of keywords according to the mean frequency of appearance. Keywords in yellow occurred with the highest frequency

Of the 100 articles in this study, the top 10 keywords are invasive thymoma, thymic carcinoma, prognosis, classification, radiotherapy, surgery, therapy, thymic epithelial tumors, myasthenia gravis, clinicopathological features, and prognostic factors. Through VOSviewer analysis, we found that thymic carcinoma, invasive thymoma, surgery, radiotherapy, and myasthenia gravis became high-density keywords (Fig. 2). Furthermore, the keywords related to basic research (growth-factor receptor, immunohistochemistry, and KIT) and immune disease (systemic-lupus-erythematosus, lichen-planus, t-cells, and red-cell aplasia) have increased in recent years.

## Discussion

Thymoma is a vital part of thymic epithelial tumors, and with continuous study, it has gradually been found to have different characteristics from other thymic epithelial tumors. Therefore, to explore the most concerned and cutting-edge hotspots in the field of thymoma research, we have constructed this bibliometric analysis specifically for thymoma, trying to sort out the current status of existing thymoma research by summarizing the most 100 cited articles on thymoma and providing a practical reference for future thymoma research. Although previous bibliometric study on thymic epithelial tumors have been published [7], our study differs from its list of top 100 highly cited articles, authors and country of publication, given the large differences between thymoma and thymic carcinoma. In addition,our study also further analyzes the keywords of included papers, hoping to help the development of future studies related to thymoma.

Original articles accounted for 3/4 (75/100) of the literature included in this study. However, it is important to note that most of these (52/75) were retrospective studies, which we believe is associated with a lower incidence of thymoma. The lower incidence makes conducting prospective studies on thymoma challenging, but 12 works are still prospective studies. The most cited article in this study was the publication by Masaoka et al., which followed 96 patients with thymoma for up to 10 years and explored the effects of staging and different treatment modalities [9]. Of the top 100 most cited works, the top 10 were original retrospective studies. Prospective studies are mainly aimed at chemoradiotherapy for thymoma. However, they were all single-arm studies, and there are studies of postoperative radiotherapy for thymoma patients who had a complete resection [10]. The study is still ongoing, and the results are worth expecting.

The United States has the highest number of published and cited in the top 100 most cited articles, reaching 41 and 6786, respectively. However, the top three most cited articles are from Japan. All of these articles were retrospective studies with large sample sizes, and all patients were followed up for a long time (up to 44 years) [9, 11, 12]. Therefore, their research results are significant for diagnosing and treating thymoma. We believe this is related to differences in the pooled incidence of thymoma in different races. For example, based on the Surveillance, Epidemiology, and End Results (SEER) database, the incidence of thymic tumors in North America is 2.14 per 1 million, and the incidence of thymic tumors in Asians (3.74 per 1 million) is higher than that of the Caucasian ethnic group (1.89 per 1 million), so thymoma in Asian populations are relatively more common. Hence, relatively large sample size studies on thymoma are also easier to conduct. We believe that slow patterns of recurrent metastasis, developments in pathological molecular techniques, and the emergence of innovative therapeutic concepts and measures have led to higher quality researches in the field of thymoma in the first decade of the twenty-first century than in other periods.

Through VOSviewer analysis, thymic carcinoma and invasive thymoma are the two keywords with higher density. Since both are attributed to thymic epithelial tumors, thymoma and thymic carcinoma are often studied together in the existing literature. For example, the International Agency for Research on Cancer (IARC) published the WHO classification of thymic epithelial tumors in March 2015, based on a multidisciplinary symposium organized by the International Thymic Malignancy Interest Group (ITMIG) in December 2011 [13]. Thymoma is an inert tumor that occurs, develops, metastasizes, and recurs more slowly. However, some thymomas have the characteristic of aggressive growth. Therefore, the treatment and prognosis of invasive thymoma have become a hot topic in thymoma research. Recent studies of invasive thymoma have focused on more minimally invasive surgical modalities [14] and treatment for neoplasm with intravascular growth [15–17].

Surgery is currently the first choice for thymoma treatment, and its position in treating thymoma and even thymic epithelial tumors has been recognized [9, 11, 18]. However, in recent years, research on the treatment of thymoma has gradually focused on radiotherapy and chemotherapy, with more prospective studies and randomized controlled trials [10, 19–21]. Currently, immunotherapy studies on thymoma have also been published, and higher PD-L1 expression was linked with a better response to treatment with immune checkpoint inhibitors but with a relatively high incidence of immune-related adverse events. Therefore, immunotherapy is not yet the standard adjuvant therapy for thymoma, and large sample size studies are required to confirm its safety.

Many studies have found that thymoma is associated with autoimmune diseases, especially myasthenia gravis. Therefore, myasthenia gravis has become one of the hot keywords in this study. In addition, immune disease-related words such as systemic-lupus-erythematosus, lichen-planus, t-cells, and red-cell aplasia also appeared in the top 100 keywords, and according to VOSviewer cluster analysis, the frequency of such words has gradually increased in recent years. Our team has also found that surgery has a certain effect on treating non-myasthenia Gravis autoimmune diseases [22]. Similarly, laboratory research on thymoma has gradually increased in recent years, and since 2000, the number of highly cited laboratory studies on thymoma has gradually increased. In total, 11 laboratory studies were included in this study, and KIT, interleukin-1alpha, PD-1/PD-L1, and titin antibodies were the hot spots. It can be seen that the research on thymoma is no longer limited to the characteristics of clinical pathology and the prognosis of treatment. The horizontal study of the association of thymoma with other diseases and the longitudinal study of the molecular gene mechanism of thymoma is receiving increasing attention.

There are several limitations to our study. First, although we used an authoritative database to identify articles, articles have different numbers of citations in different databases due to coverage differences. Therefore, there is a certain bias in the selection of literature. Second, citation analysis may not be a perfect measure of an article's impact on its field. Since the number of citations usually increases with time, the earlier articles potentially have an artificially higher impact than the more recent articles. In any case, citation analysis is still the best measurement for studies, and it can reflect the value of old articles in this field to some extent. Third, we cannot exclude self-citation from journals and authors.

## Conclusion

The first 100 most cited articles in the field of thymoma research were included in the bibliometric analysis, and a series of analyses were conducted. Most of the top 100 most cited articles are original and dominated by retrospective research. Although the most published and cited works are from the United States, the top 3 most cited articles are from Japan. Presently, the high-density keywords of thymoma research mainly come from thymic carcinoma/invasive thymoma, treatment regimen (surgery and chemoradiotherapy), immune-related diseases, and laboratory research. However, in recent years, the hot spot for thymoma research has gradually tilted towards immune-related diseases and laboratory research. This is the first bibliometric analysis of thymoma, and we hope that the findings of this study will provide guidance and inspiration for future thymoma research.

## Data Availability

Data can be provided upon request.

## Abbreviations

WOS: Web of science
IF: Impact factor
SEER: Surveillance, epidemiology, and end results
IARC: International agency for research on cancer
ITMIG: International thymic malignancy interest group
